# Perceptions of Fertility Physicians Treating Women Undergoing IVF Using an Egg Donation

**DOI:** 10.3390/ijerph19127159

**Published:** 2022-06-10

**Authors:** Reut Ben-Kimhy, Orit Taubman–Ben-Ari

**Affiliations:** 1 IVF Unit, Department of Obstetrics & Gynecology, Meir Medical Center, Kfar Saba 4428164, Israel; reutsin@hotmail.com; 2 The Gender Studies Program, Bar Ilan University, Ramat Gan 5290002, Israel; 3 The Louis and Gabi Weisfeld School of Social Work, Bar Ilan University, Ramat Gan 5290002, Israel

**Keywords:** fertility physicians, egg donation, IVF, qualitative

## Abstract

In the course of their work, medical teams are routinely exposed to difficult and stressful situations. The few studies in the literature that have examined physicians’ perceptions and responses to such situations have focused primarily on the fields of emergency medicine and chronic and terminal illness. However, the field of fertility medicine can also evoke complex feelings among physicians. The present qualitative study examined the perceptions of fertility physicians treating women undergoing egg donation. Semi-structured in-depth interviews were conducted with 20 fertility physicians, and a categorical analysis was performed. The main category to emerge was the physicians’ perception of egg donation and its implications. Two prominent themes were identified within this category: doctor–patient communication surrounding egg donation and how the idea was presented to the patient; and doctors’ perception of the implications of egg donation, including maternal identity, the relationship between mother and infant, and the mother’s sense of the child’s identity. This is the first study to consider the response to fertility treatments, a contemporary and sensitive topic, from the perspective of the physicians. The findings can contribute to physicians’ understanding of themselves and can help to devise ways to assist them in managing their emotional responses to their work for the benefit of both themselves and their patients.

## 1. Introduction

In the course of their work, medical teams are routinely exposed to harsh realities and are required to deal with stressful situations and face emotionally challenging circumstances [1], such as complex medical conditions, families in crisis, decision making under pressure, terminal illness, death, and ethical issues. Repeated exposure to physical and mental suffering can have profound and long-term effects [2], including stress [3,4] and emotional turmoil [5], which can affect the personal and family lives of caregivers and potentially impair their professional functioning [1,6]. Nevertheless, the effect of their work on the mental–emotional state of medical professionals has received little attention in the empirical literature [1,7]. The few existing studies deal with cases that involve coping with death, particularly in the field of oncology [8,9]. However, other medical conditions, although not life threatening, may also be stressful and require a complex approach on the part of the physician. One of these is infertility.

Studies show that work in the field of fertility involves situations that have the potential to evoke a range of feelings in physicians that may affect their own lives and professionalism [10,11]. Patients coping with infertility rely on the medical system to assist them [12]. Their doctors, however, are faced with a variety of challenges that relate not only to medical treatment, but also to communication and interactions between themselves and their patients [13]. The two sides maintain a complex and emotionally charged relationship that requires close cooperation between them [14]. Perceived by the patient as a problem-solving agent, the physician is expected to provide professional assistance in the decision-making process, to have in-depth knowledge of the latest fertility technologies, and at the same time to be considerate, dedicated, sensitive, and involved [15]. Physicians appear to see themselves in a similar manner [12]. Studies conducted among fertility doctors have found that communication with patients, which includes, among other things, dealing with negative emotions, particularly when they are forced to deliver bad news, is a significant source of distress [16]. Physicians may experience such news as a personal failure and disappointment, a feeling that may be triggered repeatedly given that the success of fertility treatments is still far from guaranteed [11,17]. One unwelcome piece of news is the suggestion that the couple attempt to conceive through the use of egg donation.

As a pronatalist society, Israeli women are highly motivated to become mothers and thus the pressure on physicians to provide solutions to this motivation might also be high. Given the fact that most treatments are subsidized by the government, physicians may treat women for a long period of time, sometimes even years. The possibility of egg donation, that is, the use of eggs from another woman, is raised by the doctor when a woman is unable to produce eggs of sufficient quality [18]. According to the official data of the Fertility Physicians Association in the United States (SART), in 2019, there were approximately 23,000 attempts to conceive with the help of egg or embryo donation.

The primary advantage of egg donation is that it enables a woman to become a mother, even if she does not have the biological ability to do so. Moreover, unlike adoption, for example, she also experiences pregnancy and childbirth. Pregnancy is known to play a significant role in the transition to motherhood, as it provides an opportunity for a woman to develop her maternal identity as part of the psychological preparation for motherhood [19,20]. For a woman contending with infertility, however, giving up the attempt to conceive from her own eggs and accepting the idea of donor eggs is a complex process that requires time, mental preparation, and psychological, social, and cultural adjustment [21].

Existing research in the field relates to women undergoing egg donation, with only rare attention paid to their spouses [18]. In addition, studies have examined the effects of egg donation on parenting [22,23], and on the child’s development and psychological adaptation [24,25], as well as the issue of secrecy, i.e., whether to disclose the use of a donor egg [26,27]. Still, other studies have examined the impact of the donation on the donor herself [28,29]. However, very little is known about the procedure from the point of view of reproductive physicians [30], as the critical encounter between the woman or couple and the specialist physician has not received the attention it deserves [31]. The current study, therefore, examines egg donation from the perspective of the physician, exploring their responses and perceptions of their role in this complex process.

## 2. Method

### 2.1. Participants

The sample consisted of 20 fertility physicians who treat couples wishing to conceive with the help of a donor egg and the husband’s sperm. Participants ranged in age from 44 to 67 years, with seniority ranging from 6–34 years (mean = 16.7, SD = 9.073). Four of them were women and 16 were men, 7 were managers of infertility units and 13 were senior doctors.

### 2.2. Procedure

Following approval of the Institutional Review Board, participants were recruited using criterion sampling [32], concentrating on fertility physicians working in hospitals and private clinics. They were located by contacting several doctors, who were asked to recommend additional doctors for the study. Those who agreed were contacted by the first author, who conducted all the interviews, to coordinate a time and place of their choosing for the interview where they would not be disturbed. Confidentiality was assured and the interviewee was asked to sign an informed consent form. They were told they could stop the interview at any time.

Data were collected through semi-structured, in-depth interviews, that is, they focused on the description and meaning of concrete experiences, while maintaining a flexible and dynamic format [33]. The purpose of this type of interview is to allow the interviewer to enter the world of the interviewees [32], while at the same time trying to understand the meaning they attribute to their subjective experiences [34,35]. The questions in the interview guide dealt with the physician’s perception of their role, their emotional experience of accompanying the woman or couple through treatment, and their perception of egg donation and its meaning. The researcher also had the option of adding questions or requesting clarifications spontaneously. The length of the interviews ranged from 20 to 90 min, with most lasting between 30 to 45 min.

At the end of the interview, the investigator thanked the participant for their openness, and they were offered the opportunity for a debriefing in which they could discuss any feelings the interview had aroused. In practice, none of the participants made further contact after the interview. All the interviews were recorded and transcribed. The excerpts that appear below were translated into English by a professional translator.

### 2.3. Data Analysis

The data were analyzed using Gilligan’s method [36], which involves multi-layered listening to the different levels expressed by the interviewees, first about themselves and then about the relationships and contexts within which they operate. As this method is more psychologically oriented, we felt it was most suitable for this study, which deals with the experience of the interviewees themselves and the meaning they attribute it, as well as their perceptions of the women undergoing the treatments.

Following this procedure, each of the interview transcripts was read several times, and notes, insights, and questions were recorded. The texts were then broken down into sections that were tagged as dealing with or related to a particular topic. Next, similar contents or expressions that appeared in various interviews were grouped together in an effort to determine the nature of the connections between the sections and identify the different themes. As a result of this process, broader concepts or units of meaning began to emerge, which ultimately became the common categories elicited by the interviews. By dividing the text into categories, we moved from the level of raw text to a higher level of conceptualization [37]. Each category contained several themes. In the case of the first three interviews, the analysis was conducted by both researchers independently, which enabled mutual discussion and thinking on categories and themes. Later on, the analysis was undertaken by the first author, with the second author constantly reading and reflecting on it. This was clarified in the article.

Throughout this process, the researchers consulted with each other until an agreement was reached.

## 3. Results

**Figure 1:**
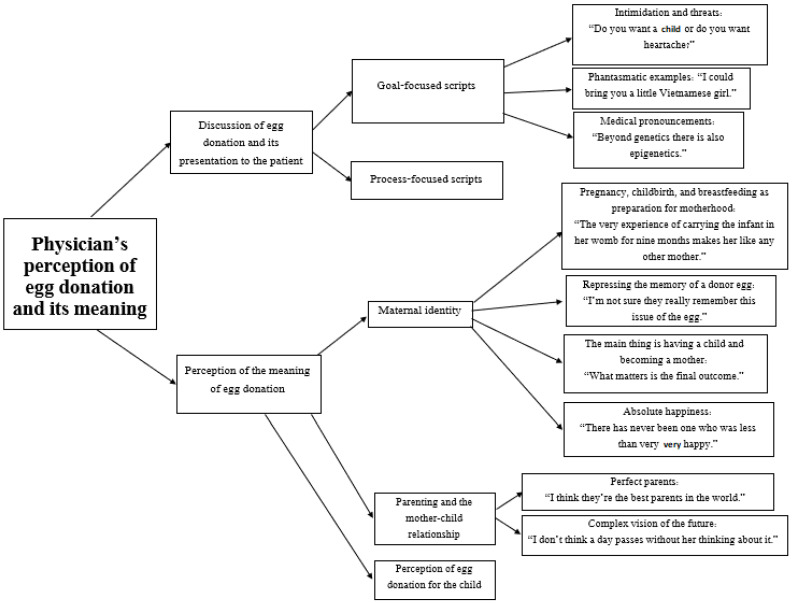
Categories and themes.

This paper reports on a prominent category that emerged from analysis of the interviews: the physician’s perception of egg donation and its meaning. The category consisted of two main themes: communication between the doctor and patient regarding the use of egg donation and how the idea is presented to the patient; and the doctor’s perception of the meaning of egg donation in relation to maternal identify, the bond between the mother and child, and the mother’s perception of the identity of the newborn (see Figure 1: categories and themes).

### 3.1. Theme 1: Discussing the Use of Egg Donation and Its Presentation to the Patient

When doctors decide that it is time to consider ceasing attempts to use the patient’s eggs and try to achieve a pregnancy through a donor egg, they wish to convey this information in the most appropriate manner. The interviews revealed that most doctors have a prepared script that they use with all patients, with only a few minor changes. As one of the doctors said:

*“I may have developed a format that I think sells the idea well...I don’t know if it sells it well, but at least I’m trying to”.*

(Interviewee 14)

The scripts employed can be divided into two groups: goal-focused and process-focused.

#### 3.1.1. Goal-Focused Scripts: “Do You Want a Child or Do You Want Treatments?”

Physicians who adopt this approach present the facts in a straightforward and one-dimensional fashion. They generate a task-oriented and goal-oriented discourse, which minimizes the possibility of reflecting on additional elements of the transition to egg donation. In other words, they relate to the practical and medical aspects of the procedure, ignoring its existentialist meaning for the patients. It is important to emphasize here the layered listening to the physicians’ expressions. Although their approach looks fundamental and dichotomous, their tone of speech was not considered by the interviewer as threatening in any way, but rather as caring and concerned. These scripts contain a variety of elements, including intimidation and threats, phantasmatic examples, and medical pronouncements.

#### 3.1.2. Intimidation and Threats: “Do You Want a Child or Do You Want Heartache?”

In order to make it unequivocally clear to the patient that there is only one way she can become a mother, some doctors make decisive statements, speaking from a position of authority as “the one who knows”. While these statements are unambiguous, they may also sound condescending.

*“I always ask them What do you want? Do you want a child or do you want heartache? Tell me what you want. Do you want to stew in your own juice, and see failure after failure after failure, or do you finally want a child? Do you want to go to a park where they ask ‘Are you the mother or the grandmother?’”*

(Interviewee 6)

*“There are women I tell very quickly, ‘Listen, this is a waste of time, accept an egg donation’. … When I see that it’s hopeless and a waste of time and a waste of the hormones they get (which we’re not sure are so healthy), I tell them, ‘Listen, do you want to be a mother before the age of about 200 or 50? It’s time to use egg donation. This is a waste of time’”.*

(Interviewee 13)

*“I tell her to look on the other side of the mountain for what she wants...whether in a year she wants to walk around IVF units doing blood tests, or she wants to walk down the street with a baby carriage. I always tell the woman, ‘OK, it’s clear, everyone wants to be healthy and rich, but your options now are either to give up on being a parent, to adopt, or to accept an egg donation, conceive, and give birth. So...which is the lesser evil? The lesser evil is egg donation’…And I tell her, ‘Listen, if that’s what you want…if your life’s dream is to be a mother, then you have to fulfill your life’s dream, like...what does it matter?...The means are less important. I don’t know…if your life’s dream is to propagate your genes, it probably won’t happen. However, if your dream is to be a mother and get pregnant, to give birth...’”*

(Interviewee 10)

#### 3.1.3. Phantasmatic Examples: “I Could Bring You a Little Vietnamese Girl”

Physicians sometimes use imaginary stories to enable the patient to connect to a scenario that they believe is similar to the idea of using egg donation in order to help the woman to accept this solution. The stories often seem to come straight out of Hollywood movies and depict the unambiguous position of absolute love for a child.

*“Sometimes I say this to women who do not agree to egg donation, I tell them, ‘Imagine for a moment, that there’s a knock on the door and you find a basket with a baby inside and a note that says, ‘I can never raise this baby. If you want it, it’s yours’. And you bring this baby into your home, a newborn baby, and.. I’m asking you now, how long will it take you to love the child as if you gave birth to it?’ She says, ‘Probably two or three days’”*.

(Interviewee 1)

*“When they come to me, they say, ‘I’ll never be able to.’ I tell them, ‘Listen, I could bring you a little two-week-old Vietnamese girl, in a year we would meet and you’d love her more than anything else in the world’”.*

(Interviewee 16)

#### 3.1.4. Medical Pronouncements: “Beyond Genetics There Is Also Epigenetics”

Some doctors adopt a purely clinical approach to support the argument for egg donation.

*“I present it as a matter of no choice. I usually give it a title, ‘What you want is a child. To get it, you need semen, you need eggs, and you need a uterus, and... hopefully, it really works like that... I‘m very happy that [in your case] the sperm is normal and the uterus is fine. Now if we get an egg you can experience pregnancy, birth, and motherhood’”.*

(Interviewee 14)

*“[I tell them] that there are other options. I simply raise the possibility. And I tell them about studies that have shown an excellent bond...I beautify the story a little and say that beyond genetics there is also epigenetics, and that this child will later love the same things that the mother loves, and he will sleep with his hand on his forehead like her...all kinds of things…everything they need to hear. And it’s also true. It’s not that I’m lying. I’m trying to show them both the positive sides...of being a younger mother instead of waiting until the age of 45 for a donation”.*

(Interview No. 11)

#### 3.1.5. Process-Focused Scripts: “There Is a Slow Process of Digesting the Information Internally”

Whereas in goal-focused scripts the emotional and cognitive aspects of the patient’s decision-making process are ignored, in process-focused scripts, the physician relates to the procedure from the point of view of the patient as well. Here, the doctor displays an understanding of the complexity of the decision and the need for time to digest the idea.

*“I also tell everyone that it’s…I warn them that it’s hard to digest, that they need to go home, and when she’s convinced...by me or by another doctor, they will come to internalize it. But it’s a process. I don’t remember anyone who, at the end, wasn’t open to it. There’s a time of internal processing, of slowly getting used to it and digesting it, but eventually everyone accepts it... Usually, I think at some point I try to be more decisive and less vague. Also, I usually say, ‘Go home, sleep on it, read about it...even reading about it on the internet is okay...anywhere. Internalize it, ask questions if you have any, but...there’s no point in continuing like this’”.*

(Interviewee 14)

*“I always advise them. I tell them all the options, including egg donation, so as not to miss the pregnancy train either... At first, almost no couple agrees. Almost. It takes a few treatments until they understand that this is what needs to be done... So I suggest, but I don’t press. I mean if she wants another treatment and another treatment. But then I try to set a goal with her. ‘So let’s decide. Let’s decide that you undergo another treatment or two and if that doesn’t work, we move on?’ Then it is easier for her…I always tell her, ‘You have to get to the point where you feel there was nothing you didn’t try in your desire to have a child. When we get to that point, it will be ok. However, we should try and set a clear timeline for that’”.*

(Interviewee 8)

*“I do it gradually...I tell her, ‘You know there are all kinds of options, you know that if you lived in the US, you could not do 10 IVF treatments. They would probably suggest egg donation after two or three alternative treatments’... I never come right out and say...‘Listen to me’....That’s an awful kick in the stomach… it... also, how can you continue after that, it’s an expression of total lack of faith in the woman, so you too... from that moment on, you become a person they don’t believe in at all anymore. How can you continue to give her those treatments if you’ve declared that... So I say there are all kinds of options, some do surrogacy, and some do this... it’s one of the options... In most cases I’ll tell her, look you can do follow-ups and this and that... I also see her response, because, look, most come prepared… If I see that she’s not ready, I tell her, let’s do a month or two of follow-ups on your health, come as if… And then with her own eyes she sees she doesn’t have the follicles. It makes it much easier... Like if you do a PGS for a woman, check her embryos and she sees that each one is chromosomally abnormal, it makes it very easy for her, she says ok, let’s move on to the egg donation”.*

(Interviewee 16)

### 3.2. Theme 2: The Doctor’s Perception of the Meaning of Egg Donation

All the physicians interviewed, without exception, believed that egg donation is an excellent solution for a woman who wants to be a mother and cannot conceive with her own eggs. Their discussion with the patients, therefore, relates to the woman’s expectations of having a child, rather than to her expectations of having a child who shares her genes. The possible duality of the mother loving a child conceived by means of a donor egg, while at the same time mourning the fact that it is not the child she fantasized about, was mentioned only marginally. Moreover, there appears to be an almost paradoxical split between the doctors’ perception of the procedure from the point of view of the woman, that is, her maternal identity, her parenting, and her relationship with the baby, and from the point of view of the child.

#### 3.2.1. Maternal Identity

In the doctors’ discourse in support of the use of egg donation, the donor is totally absent, as is the future baby. Instead, the physicians focus on the procedure as the best solution for achieving the woman’s goal of pregnancy, childbirth, and motherhood. Four different types of arguments emerged from the interviews.

#### 3.2.2. Pregnancy, Childbirth and Breastfeeding as Preparation for Motherhood: “The Very Experience of Carrying the Infant in Her Womb for Nine Months Makes Her Like Any Other Mother”

Some doctors contend that the fact that the baby grows in the woman’s womb will help her to feel maternal love. In other words, carrying the baby, giving birth, and later breastfeeding enables the patient to undergo a process at the end of which she will feel that the child is entirely hers, just as any other child.

*“The very experience of carrying the infant in her womb for nine months makes her like any other mother. It’s probably different from adoption when suddenly a new child lands in the home. No, here there are expectations, and there’s a date and she feels the baby moving, and ultrasounds, and good and not good... I think in terms of preparation it’s just like any other mother. It doesn’t seem different to me”.*

(Interviewee 1)

*“It’s something very abstract, this part. I mean that this is an egg from someone else. I still think that the bonding element, in my opinion, is the 9 months in the womb, and the mutual experiences of birth, and breastfeeding, and the first year after the birth. It seems to me that this is the important part. And it’s something abstract. Think of yourself. Somebody tells you you’re actually your father’s and your mother’s child and you’ve been with her for nine months but the egg is someone else’s. Like, so what? You are you. and you won’t suddenly love your mother less”.*

(Interviewee 14)

#### 3.2.3. Repressing the Memory of a Donor Egg: “I’m Not Sure They Really Remember This Issue of the Egg”

Other doctors believe that the pregnancy and the baby have the power to erase from memory the use of egg donation, leaving only the desirable aspect of the process: motherhood.

*“Once the pregnancy begins, they forget…I’ve been in the field for 20 years, and I don’t remember anyone who came and said ‘I regret that I accepted an egg donation.’ I don’t remember a single one like that”.*

(Interviewee 5)

*“I always think to myself, I’m not sure they really remember this issue of the egg. I think once they have a baby to raise, it’s not so important. It’s not there anymore. Life keeps carrying them forward and they have so much to do with this child...to bring it up and educate it”.*

(Interviewee 6)

*“I don’t have the tools of a psychologist, but when you see a heartbeat on the ultrasound, you’re already at a point when it would be very interesting to see if there’s any difference between a pregnancy from a donor egg and one that isn’t. In my opinion, there’s no difference. From that moment, it all comes together and a pregnancy begins, a full pregnancy, regardless of how it began. Perhaps, subconsciously they tend to think, ‘Maybe it was from my egg after all and it’s impossible to know.’ I don’t know. And it doesn’t matter how the child looks afterward, who it looks like, if anyone. It always resembles someone in the family when they were young. ‘That’s exactly what I looked like’”.*

(Interviewee 14)

*“A woman who accepts a donor egg, as soon as she makes the switch in her head and moves on, she’s the happiest mother in the world. They have a child, they’re happy, they’ve already forgotten where the egg came from. They want a child, a healthy child”.*

(Interviewee 17)

#### 3.2.4. The Main Thing Is Having a Child and Becoming a Mother: “What Matters Is the Final Outcome”

Many doctors regard only the final outcome as significant. When pregnancy is achieved through egg donation, everything else, including the patient’s thoughts and feelings about the procedure and the many failures she experienced along the way, are pushed aside and become irrelevant in light of the current success.

*“I think what matters is the final outcome and if the final outcome is good and it ultimately fulfills the dream of parenting and raising children, then even if it happened late and even if it took a little longer than expected and even if it wasn’t in the usual way, I think that’s what matters”.*

(Interviewee 5)

*“Whoever arrives at egg donation, arrives at peace of mind. That’s how I see it. It’s a kind of safe haven. Because this whole war, what is it for? A child. For the child. It is all for the child. It is not about being pregnant. It is not about having a big belly, it’s for a child”.*

(Interviewee 6)

#### 3.2.5. Absolute Happiness: “There Has Never Been One Who Was Less Than Very, Very Happy”

Some physicians relate to only one emotion, happiness, regarding it as exclusively descriptive of the patient’s feelings about the pregnancy and baby.

*“My feeling is…if I have to quantify the happiness of the patients, I don’t see any difference in the happiness, say, of a patient who uses egg donation or sperm donation compared to the happiness of other patients. My impression is that their happiness…from the child is very similar. I see completeness and I see joy...and women who come and say ‘This is the best thing I’ve ever done in my life’”.*

(Interviewee 1)

*“Now, I have a lot [of patients] who are single parents, I mean they need both an egg donation and a sperm donation… They’re happy!”*

(Interviewee 13)

*“I tell you as someone who has accompanied hundreds of women, there has never been one who has been less than very, very happy.”*

(Interviewee 16)

*“I have a lot of single women who have succeeding in having a child from their own eggs and a sperm donation. Then they come and say to me, ‘Listen, if I didn’t have a child with my egg and a sperm donation, then I would go through the process of sperm donation and egg donation. But now I have one child from my egg and a donor’s sperm, and on the other hand I’ll have a child from a donor egg and a donor’s sperm. Then nothing will be mine. And I’m afraid that I will love the child from the egg and sperm donation less than the child that came from my egg. That’s not right!’ But after that they turn around and they love the child with their whole heart... and they’re all the happiest women in the world”.*

(Interviewee 5)

### 3.3. Parenting and the Mother–Infant Bond

Another angle from which doctors look at egg donation relates to the bond between mother and baby. Here, too, there were several approaches to this issue.

#### 3.3.1. Perfect Parents: “I Think They’re the Best Parents in the World”

Most physicians described parenting and the relationship between the mother and the baby born from a donor egg as no different from parenting a biological child, and perhaps even better.

*“It’s like they’re amazing parents...They love their children, really love them”.*

(Interviewee 3)

*“I think they’re the best parents in the world. The children come from deep desire, expectation, choice. It’s not like my mother and father were about to get a divorce and suddenly they got pregnant... and my mother wasn’t even sure he was my father. Then they debated whether to get rid of me or keep me… and they said, fine, come on, we’ll keep you and I was born and they immediately divorced and you should know we didn’t want you and even thought about getting rid of you, but in the end, we kept you, you were born. In contrast, your mother wanted the most wonderful child in the world, and she didn’t have a partner, so she went to the sperm bank, and chose the best sperm, and she came and did inseminations here. It wasn’t some...one-night stand. Then she needed an egg from the Medical Center in Herzliya or from the Ukraine, and put them together. She had to work for it!”*

(Interviewee 5)

#### 3.3.2. Complex Vision of the Future: “I Don’t Think a Day Passes without Her Thinking about It”

Among the plethora of voices, several doctors painted a more complex picture of the emotions and ambivalence that may arise as a result of egg donation.

*“Afterwards, parenting…I think it’s…you know, it will always stay with her. I don’t think it makes her a better or worse mother.... I don’t think a day passes without her thinking about it. That’s my opinion. But on the other hand, there are 24 h in a day, and for 23.5 of them, you’re happy because you have a family and more than one child, so I think we did something. We did something”.*

(Interviewee 9)

*“Ask couples who have adopted and have a biological child, they won’t understand what you’re talking about. Ask her how she feels....I’m not going to tell you that there were no people who said,...Look…you know there is always a feeling of having missed the chance, people usually waited and waited....Look…as soon as you go to the doctor at the age of 45 for treatments it brings your whole life up, all the guys who wanted to marry you, or that you imagined wanted to marry you, or your mom imagined it. I mean, it brings it all up, the 30 years of failure. So it’s obvious that with an egg donation as well, it could be that in another 10 years, she’ll sit in the park and say, ‘I wonder if I’d married at the age of 20, what my grandchildren running around here now would look like, something like that’”.*

(Interviewee 16)

#### 3.3.3. Perceptions of Egg Donation from the Point of View of the Child

Although all the physicians presented egg donation as an excellent solution for the mother, other voices were heard when they addressed the procedure from the perspective of the future child. In this context, a complexity emerged, with doctors expressing anxieties and feelings of pity and compassion for the difficulty involved in the child’s eventual discovery that they are not genetically related to their mother.

*“I wouldn’t want my mother to come to me one day and tell me, just to let you know, you’re not my biological child, because I don’t know what that means… What I could understand from her telling me that is that she doesn’t really love me… It would be better not to tell me such things. Even when people tell me bad things, I say keep it to yourself, what do I need your problems for... For a child it’s pretty bad... Like children get anxious. If she’s not my biological mother, and I just quarreled with her about the scooter, will she throw me out of the house?”*

(Interviewee 16)

*“Let’s say that with life and maturity and defense and repression mechanisms and everything else they are...yes, they’ll learn how to live with...it and make it... For them it will fade. It will become less significant. But the child won’t have…they won’t be able to process it. I think it’s awfully hard for a child! I really feel sorry for these children”.*

(Interviewee 4)

*“Biologically, there’s a small problem with it. If genetic problems develop later, and they find out they’re not the child of their mother, okay? It raises some doubts in the corner of your mind. But if this isn’t the case, I don’t think it’s good for them....It’s like if someone tells you today that you are not your mother’s daughter, what would that do to you? Upheaval. It would shake you to the core, and why do that to you? So, if there’s a genetic reason, then maybe it’s worth [telling you]. Again, this is a question that has to be dealt with”.*

(Interviewee 6)

It appears to be quite difficult for the physician to contend with the complexity. Considering all the perspectives, including their own position, the patient, and the child, as well as the perspective of the donor, the spouse, and the sociocultural and economic implications, might create an internal conflict that could affect their emotional state. As one of the doctors put it:

*“With egg donation I know the chance is better, so I feel good about it. On the other hand, with egg donation there is always the... From my point of view it’s problematic. Because if I perform an egg donation for someone who has no eggs at 40, it’s much easier for me, because I feel it’s legitimate. When I perform an egg donation at an older age...there’s an ethical problem with it. The age. The mother is old. That means the child will grow up in a home with elderly parents… The moment I do this knowingly, in this way, it’s a little hard sometimes. You make a dream come true for them, but you forget that you’re putting someone in the equation who is…who will be born into a difficult reality”.*

(Interviewee 12)

To conclude, fertility physicians who perform egg donation are compelled to contend with emotional issues alongside medical considerations. This aspect of treatment is dealt with both internally and with their patients, as part of their interactions with them.

## 4. Discussion

In the course of fertility treatments, at some point the physician may believe it is time to cease the attempts to enable the woman to conceive from her own eggs and move on to the use of egg donation. This is a significant crossroads in many ways, as it has considerable medical, economic, and emotional implications.

All the physicians interviewed in the present study, without exception, believed that egg donation is an excellent solution for a woman who wants to be a mother and cannot conceive genetically. Indeed, previous studies have shown that women who chose egg donation did not regret their decision [38] and derived a strong sense of satisfaction from parenting [39]. Nevertheless, while for the physician egg donation is an opportunity to create a new life, for the patient it also entails a sense of loss and finality [40,41]. Whereas the medical profession focuses on the rising success rates resulting from the transition to the use of eggs from a young and healthy donor, for a woman, the need to use a donor egg is another failure in a long line of unsuccessful attempts to become a mother [42]. In other words, there is a gap between the pragmatic, one-dimensional medical perception of the procedure and the woman’s emotional response, which is generally more complex, including both a sense of success and joy when she ultimately gives birth, and feelings of failure and grief for the biological baby she will never have. Most of the interviews in this study indicate that the discussion that doctors have with patients about this option relates to the woman’s hope for a child per se, and not to her hope for a child who shares her genes. It seems that the possibility that the mother can love a child conceived from a donor egg and at the same time mourn the fact that it is not the child she fantasized about is addressed only marginally by the doctors both as reflected in the interviews and in their communications with the women.

Thus, while the physician and patient are working together to achieve the same goal, their emotional responses and perceptions of reality may differ.

In respect to the manner in which the physicians present the idea of egg donation to their patients, it was found that most physicians do so unequivocally and one-dimensionally. They describe a discourse that is linear, task-oriented or goal-oriented, and focuses on the bottom line. In addition, they employ various strategies to explain the benefits of the solution, breaking it down into its most basic components and using specific, concrete, and dichotomous questions that the patient might find easier to answer, such as “Do you want to be a mother or not?”. In addition, they use examples resembling Hollywood scripts, depicting only the happy end, and cite medical information in support of the option, ignoring other concerns the patient might have, including the losses (real and abstract) that the process entails, the fear of the unknown, doubts about the procedure, the potential change in the meaning of “family,” and so on.

The literature shows that the physician influences the way the patient feels about his or her illness and its treatment [43]. Thus, the doctor’s opinion plays a significant role in the patient’s medical decisions. Consequently, the way in which the doctor presents the information is extremely important. Moreover, the opinions and professional decisions of physicians may be influenced by non-medical factors, including their own personal values and priorities and their perception of patient characteristics, such as age and life circumstances [44]. Furthermore, the influence of social values must also be considered. Israel is a strongly familial society in which the “natural” role of motherhood is deeply embedded in the culture and has even been “nationalized”, that is, regarded as a civic duty [45]. This context may also have played a role in the physicians’ determination to turn the woman into a mother at all costs, even if not a biological mother. Indeed, as noted above, all the interviewees, without exception, were in favor of egg donation as a means of realizing motherhood in what they believed to be the fastest and most effective way.

This may explain why physicians, who, for whatever reasons, believe it is in their patients’ best interest to switch to egg donation, provide them with information subjectively, focusing only on the benefits and ignoring the negative. Previous studies in the field of fertility have also found that physicians tend to provide information about the success of treatments and physical risks, while omitting from the discourse the possibility of unsuccessful pregnancies and the emotional consequences [46].

Providing incomplete information can have long-term consequences for the patient. Studies dealing with the ability to make medical decisions have shown that conveying broad information about the treatment and its possible consequences helps to minimize conflict and remorse after the decision [47]. In the case of egg donation, it has been found that most women do not regret their decision and display optimal and happy parenting. Nevertheless, it is possible that by expressing a firm and unequivocal position, doctors do not give themselves the opportunity to listen to the patient’s authentic feelings. It has therefore been recommended that fertility physicians inform the patient about the possible medical complications of egg donation and at the same time relate to the emotional consequences, thereby helping her to reach an autonomous decision by examining her motives for choosing this procedure and adjusting her feelings, beliefs, and expectations to reality [46], as well as balancing the benefits and costs to her physical and mental health [38]. In addition, even before expressing their opinion, physicians should understand patients’ values and preferences regarding their desired family configuration [47]. The physician’s support in the decision-making process is important, since ultimately most patients prefer to reach a joint decision with the medical professional, rather than leaving it up to the physician or deciding for themselves [48].

In the present study, it was found that the doctors regard only the final product as significant. Their discourse, therefore, focuses solely on egg donation as the way to achieve motherhood in the conviction that pregnancy and childbirth are the essential characteristics of being a mother. Some believe that this experience itself has the power to erase the past, particularly the fact that the baby was the result of egg donation. Consequently, they stress one emotion, happiness, considering it to exclusively define the patient’s feelings, as any other thoughts and feelings, as well as the previous history of failures, will become irrelevant as soon as the procedure succeeds.

Studies examining patients’ perceptions of egg donation have found that some women similarly display a desire to ignore anything that obscures the happiness of the end result in order to preserve the fantasy of “normal” motherhood. Studies have shown denial behaviors and defenses against the fact that the pregnancy was achieved through the use of another woman’s egg [49].

It is important to note that there is very little information about mothers’ attitudes to a child conceived from a donor egg, and the results of existing research are not consistent. Some studies have shown that the egg donation did not affect the mother’s relationship with the newborn [22,49,50,51,52], and in others, women reported feeling an emotional connection to the fetus in their womb, a bond that persisted after the birth [24]. On the other hand, it has been found that the egg donation aroused ambivalence and emotional complexity [53], and that during the pregnancy, women were concerned about whether they would feel as a “real mother” to their child [41].

Imrie and colleagues [40] conducted a qualitative study that examined women’s thoughts and feelings about their relationship with their children who were conceived from a donor egg. It was found that the women employed several strategies that are similar to those displayed here by physicians to help to define their maternal identity. The researchers report that the process of “making the child their own” began before conception and continued throughout the first years of the infant’s life. Among the similar strategies identified were minimizing the significance of the donation and drawing a line between the egg and the baby, focusing on the future child and the woman’s strong desire for it, forgetting the donor’s existence during pregnancy, and treating pregnancy as a process that prepares her to see the baby as her own. Tsui and Cheng [38], who examined the feelings of women who used sperm or egg donation, found that pregnancy was a transition to motherhood. The mothers described the process of carrying the fetus in their womb as helping to forge a bond between themselves and the baby, despite the lack of a genetic connection.

Moreover, in contrast to the discourse of most of the physicians in the current study, which presents egg donation as solely positive, studies of patients reveal ambivalence and complexity, with women continuing to be concerned by the non-genetic relationship with the child, particularly during pregnancy. Even after birth, women were found to be very happy with motherhood and grateful to the donor, while at the same time expressing uncertainty and anxiety over being the mother of a child conceived from a donor egg [49]. In other words, whereas most physicians held a dichotomous “either–or” position, the patients’ attitude to egg donation and its implications appears to be more complex and multi-faceted.

It should be noted that a number of physicians in the current study were able to recognize the complexity of the situation and include additional emotional and cognitive components in their discourse. Theoretically, this approach should enable them to view reality, and their role in it, more clearly.

Another issue addressed in the present study is the bond between the mother and her baby. Most doctors described this relationship as no different from parenting a biological child, and perhaps even better. However, some physicians painted a more complex picture, relating to other emotions that might color this relationship. The complexity seemed to emerge when doctors looked at egg donation from the patient’s perspective and not their own, and consequently became aware of the emotional dilemma. A similar pattern can be seen in previous studies examining the quality and mode of parenthood and the relationship between mothers and children of different ages who were conceived by means of egg donation. Most studies found a positive parent–child relationship, with some even indicating a better-than-genetic relationship [22,51,52]. In contrast, several studies reveal the ambivalence and complexity aroused by egg donation [41], and the differences between parenting a child from a donor egg versus a biological child [23].

Interestingly, despite the physicians’ determination to present egg donation as an excellent solution for the mother, they were less equivocal when they considered the procedure from the perspective of the future child. In this context, they expressed concerns and compassion for the difficulty involved in the child’s discovery that they are not genetically related to their mother. Thus, physicians appear to hold a paradoxical view, seeing the positive effect of egg donation on the patient, and the potential negative impact on the child.

It is possible that, in order to be able to perform their professional role faithfully and offer women the option of egg donation as a solution to their infertility problem, doctors must, whether consciously or unconsciously, ignore the negative factors in the equation. Vivid awareness of the potential difficulties and harm to the child might make it hard for them to suggest the procedure to their patients. They therefore regard, and present, egg donation as exclusively beneficial, enabling them greater peace of mind in their professional capacity.

The doctors’ focus on achieving pregnancy, whatever the means, might also be related to the cultural climate in Israel, which sanctifies motherhood, even when it comes at a cost, and even when it may not be in the best interest of the future child. This attitude is particularly compelling when there is little moral dilemma, which is true for most women undergoing fertility treatment. It may be more difficult to justify when there are greater moral issues involved, such as in the case of a woman who is older or suffers from a serious underlying illness or a couple with mental disorders.

To conclude, maintaining the position that egg donation is an excellent solution for women in fertility treatment, while ignoring the potentially negative implications of the procedure, appears to protect the physician from any discomfort they might arouse. However, this approach does not allow the doctor to see the whole picture and consider all the aspects that are relevant to the woman in making her decision. This one-dimensional vision misses out on the inherent complexity and may seem to disregard the patient’s emotional response. Nonetheless, it is evident from previous research that, at some point in the process, patients may also adopt a similar perspective to help them to continue on their journey toward their desired goal [40]. That is, along the long road to motherhood, there are both similarities and differences in the way physicians and patients deal with the complex issue of egg donation, which is in any case difficult for both parties. Consequently, they may use similar mechanisms of symbiosis, independence, and relying on a fantasy in order to arrive at a happy conclusion. 

### 4.1. Limitations

This study examined the perceptions of fertility doctors suggesting egg donation to women and couples attempting to conceive. Certain limitations should be noted. First, this is a qualitative study that employed a non-representative sample of 20 male and female physicians, and therefore the results cannot be generalized to the entire population of fertility physicians. This limitation is common to most qualitative studies, which typically use a relatively small sample.

In addition, the study was conducted in Israel, which is a familyist and pronatalist society that encourages childbirth, with parenthood being a central value and even considered virtually a social duty. As a result, fertility treatments in Israel are largely government-funded, so that women may undergo a large number of treatments before considering the use of egg donation. The intensive investment in childbirth, the long and arduous process of treatment, and the place of the fertility doctor in the lives of women and couples dealing with difficulty conceiving may make Israel different from other countries in which motherhood is not of paramount value and fertility treatments are not subsidized. Further studies conducted in other societies are needed to determine whether or to what extent our findings are culture-bound.

### 4.2. Implications of the Study

This study has both theoretical and practical implications. On the theoretical level, it places at the forefront the population of physicians, which, although extremely significant in the realm of fertility treatment, has yet to receive adequate research attention. It was found in this study that the process and its results both affect and are affected by the doctors who accompany the women and couples undergoing treatment. Moreover, the study contributes to the increasing awareness of the complexity of the healthcare provider’s experience, and reenforces the need for physicians to consider the whole picture from the point of view of the patient.

On the practical level, the findings indicate that fertility physicians see themselves as supportive of their patients, while encouraging and containing them in moments of distress. This role can put a heavy weight on their shoulders. At the same time, they also have to deal with their own feelings, such as personal failure and futility when treatment is unsuccessful. It might therefore be beneficial to offer fertility physicians psychological counseling to help them to process their feelings. Indeed, it has been found that physicians who process their emotions are less prone to professional burnout [54].

Such counseling could also help to raise physicians’ awareness of the way in which they communicate with their patients. For example, it was found in this study that non-medical factors, such as personal values, perceptions, and priorities, also influence the doctors’ professional opinions and decisions. Understanding the impact of these factors may help them to control and choose their approach to the patient, the information they convey, among others. Furthermore, it could help them to integrate their perceptions of the mother and of the child, enabling them to continue to help women to conceive with the aid of egg donation without experiencing conflicting emotions. This would undoubtedly be of benefit not only to the physician, but also to their patients, who are faced with the need to make a decision about their future and choices.

### 4.3. Suggestions for Further Research

The present study can provide a basis for further research, both qualitative and quantitative, in the field of infertility from the point of view of the medical staff. Future studies might examine additional professionals, such as nurses and therapists, as well as physicians’ attitudes to other fertility issues, including surrogacy, co-parenting, and single parenting. In addition, a study examining women and couples who conceived by means of egg donation at later stages in their parenting could shed further light on the implications of this procedure.

## 5. Conclusions

The field of fertility medicine has the potential to evoke complex feelings among physicians. However, such feelings have seldom been studied. The findings of the current study indicate that maintaining the position that egg donation is an excellent solution for women in fertility treatment, while ignoring the potentially negative implications of the procedure, psychologically protect the physicians. However, this approach may hinder the doctor from seeing the whole picture and consider all the relevant aspects to the woman in making her decision. This one-dimensional vision misses out on the inherent complexity and disregards the patient’s emotional response. Nonetheless, at some point in the process, patients may also adopt a similar perspective to help them to continue on their journey toward their desired goal of having a child. Thus, although at certain times doctors and patients may seem to be on different sides, in others they see the complexity eye to eye. Consequently, they may use similar mechanisms to arrive at the final conclusion. In view of these findings, and the sparse empirical knowledge available, there is a need for further investigations to shed more light on the feelings and perceptions of fertility doctors and identify the gaps between them and their patients in the effort to achieve motherhood.

## Data Availability

The dataset analyzed during the current study is available from the corresponding author upon reasonable request.
